# Epidemiology and practice patterns for male breast cancer compared with female breast cancer in Japan

**DOI:** 10.1002/cam4.3267

**Published:** 2020-07-01

**Authors:** Taisuke Ishii, Eriko Nakano, Tomone Watanabe, Takahiro Higashi

**Affiliations:** ^1^ Division of Health Services Research National Cancer Center Tokyo Japan; ^2^ Department of Medical Oncology St. Luke’s International Hospital Tokyo Japan

**Keywords:** breast neoplasms, breast neoplasms, male, medical records, registries, therapeutics

## Abstract

**Background:**

The incidence of male breast cancer (MBC), although rare, has shown an increase. However, the current epidemiology of and practice patterns for MBC remain unclear. This study evaluated the characteristics and care patterns for MBC compared with female breast cancer (FBC) in Japan.

**Methods:**

Using the National Database of Hospital‐Based Cancer Registries (HBCR) linked to the Diagnosis Procedure Combination data, we analyzed newly diagnosed breast cancer cases between January 2012 and December 2015 at participating hospitals in a large quality‐of‐care monitoring project. We employed logistic regression models to assess cancer treatment differences between MBC and FBC in patients who were indicated for adjuvant radiation therapy and neo‐adjuvant/adjuvant chemotherapy.

**Results:**

Of 142,636 breast cancer patients, 870 (0.61%) were MBC patients. At diagnosis, the mean age of MBC patients was 10 years older than FBC patients (70 *vs* 60 years; *P* < .001). Advanced‐stage cancer was more frequently observed in MBC than in FBC (stage III/IV 18.9%/6.1% vs 10.6%/5.2%). Despite this, MBC patients were less likely to receive adjuvant radiation therapy and neo‐adjuvant/adjuvant chemotherapy. Gender was an independent treatment determinant factor for chemotherapy decisions.

**Conclusion:**

MBC patients were older and had higher stages of cancer than FBC patients at diagnosis, but received suboptimal treatment.

## Graphical abstract

Male breast cancer was rare cancer and diagnosed with more advanced stage than female breast cancer. However, male breast cancer patients were received suboptimal adjuvant therapy, including radiation and chemotherapy despite of guidelines which recommend the same indication and regimen to both male and female breast cancer.

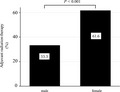

## 1 INTRODUCTION

Male breast cancer (MBC) is rare, representing fewer than 1% of all breast cancer in the United States^1^; however, recent trends show increased incidence.^2^ Epidemiological differences in male and female breast cancer (FBC), such as an older age and a higher stage at diagnosis in MBC patients have been noted. Additionally, there is a higher chance of MBC being hormone receptor‐positive and a lower chance of exhibiting human epidermal growth factor receptor‐2 (HER2) overexpression.^2^, ^3^, ^4^, ^5^ Consequently, cancer treatment differs between MBC and FBC patients. A study citing the National Cancer Data Base data in the United States from 1985 to 1994 showed that MBC patients were more likely to receive mastectomy and radiation therapy after surgery and were less likely to receive adjuvant chemotherapy.^6^ A recent study showed that mortality rates of both MBC and FBC patients have improved in the last 20 years,^7^ which may be attributed to advances in chemotherapy and adoption of new technologies.

The decades old practice pattern studies currently used for comparing MBC and FBC might not reflect the current status of care. To the best of our knowledge, there have been no recent studies using large datasets to describe practice pattern for MBC, and identify the differences from FBC. The National Comprehensive Cancer Network (NCCN) guidelines in the United States and Japanese Breast Cancer Society Guidelines contain similar recommendations, such as the indications for adjuvant radiation therapy and adjuvant systemic therapy for both MBC and FBC patients. However, the epidemiology of and current practices for MBC remain largely unknown. Therefore, the present study aimed to close the information gap and characterize the epidemiology and patterns of care for MBC compared to those for FBC.

## 2 METHODS

### 2.1 Study population

The study included all breast cancer patients who were newly diagnosed from January 2012 to December 2015 at hospitals that participated in a large quality‐of‐care monitoring project.^8^ Details of the data collection process are described elsewhere.^9^ The project aimed to monitor cancer patients’ quality of care and collected health insurance claims‐equivalent data from the Diagnosis Procedure Combination (DPC) survey in a manner that could be linked to the National Database of Hospital‐Based Cancer Registries (HBCR). The DPC survey data are not health insurance claims per se, but comprise data on all health services provided to the patients that would have been submitted as insurance claims under the fee‐for‐service reimbursement. The HBCR is a mandatory cancer incidence reporting system for cancer care hospitals designated by the Ministry of Health, Labour and Welfare in Japan. It is also optionally operated in voluntarily participating, nondesignated hospitals that play similar roles in their respective communities. Hospitals that participate in the HBCR, both designated hospitals and others, were invited to participate in the quality‐of‐care monitoring project. This study was reviewed and approved by the institutional review board of the National Cancer Center in Japan.

The HBCR data include clinical and patient information, such as age and gender, clinical and pathological stages of cancer, tumor‐node‐metastasis (TNM) classifications, tumor location, and histopathological type according to the International Classification of Diseases Oncology 3rd edition (ICD‐O‐3). All breast cancer patients in the database were included in this analysis. The DPC survey data contains coded information on all billable health services provided by the facility in a similar fashion as the insurance claims. We collected DPC survey data for patients registered in the HBCR database from the October of the previous year of diagnosis to the end of the following year of diagnosis (eg, for patients diagnosed in 2012 and October 2011 to December 2013) and linked them to the HBCR database.

We used combined stages because pathological stages were only reported for resected tumors. We created a combined stage combining the clinical and pathological stages according to Union for International Cancer Control 7th edition. For the combined stage, we used pathological stage. In the absence of this, we used the clinical stage.^10^, ^11^ Combined stages represented the most accurate stage of the diseases. We included all stages from stage 0 to IV.

### 2.2 Statistical analysis

We described MBC epidemiology and performed a comparative analysis of practice patterns between MBC and FBC. Along with overall sample analysis, we performed separate analyses for two groups of patients — stage 0–III and stage IV cancer — because recommended therapies are different for these two groups. In the stage 0–III patient group, we further limited our analysis to those patients who were candidates for breast surgery, as well as adjuvant therapies using radiation, hormone, chemotherapy, and trastuzumab. Because the HBCR did not contain data on hormone receptor and HER2 overexpression status, we used combined stages and TNM to determine the candidates for adjuvant therapies using radiation, chemotherapy, hormone, and trastuzumab. To assess differences in practice concerning adjuvant radiation therapy, we focused on postsurgical stage 0–III patients who had a tumor size ≥5 cm, or ≥4 positive axillary lymph nodes after mastectomy, or had undergone lumpectomy because these patients are the candidate for adjuvant radiation therapy according to the clinical guidelines and previous studies.^12^, ^13^, ^14^, ^15^, ^16^, ^17^ In the assessment of adjuvant hormone therapy, we focused on post‐surgical stage 0–III patients. Finally, in the assessment of adjuvant chemotherapy or adjuvant trastuzumab therapy, we focused on stage II or stage III postsurgical patients, because these patients were recommended to receive neoadjuvant/adjuvant chemotherapy in clinical guidelines.^17^, ^18^ Also, we defined these patients as candidate patients for adjuvant radiation therapy and neoadjuvant/adjuvant chemotherapy in this study. Student's t‐test was used to compare continuous variables and the chi‐square test was used to compare categorical variables between MBC and FBC patients. Differences in the use of adjuvant radiation therapy, neo‐adjuvant/adjuvant chemotherapy, and chemotherapy for stage IV patients were further analyzed using logistic regression models after adjusting for other factors. For adjuvant radiation therapy, we adjusted for age at diagnosis, combined stage, and the surgical procedure used to treat the breast cancer (mastectomy or lumpectomy). The likelihood of receiving neo‐adjuvant/adjuvant chemotherapy between genders, after adjusting for age at diagnosis and combined stage, was compared. Lastly, considering the decision to perform chemotherapy for stage IV patients, we adjusted for age at diagnosis. We defined adjuvant radiation therapy as postoperative radiation therapy within 140 days (if adjuvant chemotherapy was not performed) or 240 days (if adjuvant chemotherapy was performed) after surgery. We defined neo‐adjuvant chemotherapy as preoperative chemotherapy initiated within 200 days preoperatively and adjuvant chemotherapy as postoperative chemotherapy initiated within 100 days postoperatively for candidate patients.

All statistical analyses were two‐sided and performed as part of the quality‐of‐care/practice pattern project that was approved by the institutional review board of the National Cancer Center, Japan. Statistical analyses were performed using Stata version 13.2 (StataCorp LP, College Station, TX, USA). A *P*‐value < .05 indicated significance.

## 3 RESULTS

### 3.1 Patient characteristics

A total of 143 190 patients were diagnosed with breast cancer between January 2012 and December 2015 at 312 designated cancer care hospitals and 177 nondesignated hospitals. Of these, 550 were excluded because of unknown stage and four were excluded because their age at diagnosis was <20 years. Of the remaining 142,636 patients, 870 (0.61%) were MBC patients, whose mean age at diagnosis was 70 years (SD 12), and that of FBC patients was 60 years (SD 14) (*P* < .001; Table 1). The number of MBC patients with stage IV (6.1% *vs* 5.2%; *P* = .18) and stage III (18.9% *vs* 10.6%) cancers was slightly higher than the number of FBC patients.

**Table 1:** Characteristics of and treatments for all stage cancer patients

Characteristics	Total (N = 142 636)	Male (N = 870)	Female (N = 141 766)	*P*‐value
Age, mean (SD, min–max), years	60 (14, 20‐105)	70 (12, 27‐95)	60 (14, 20‐105)	<.001
≤64, n (%)	87 530 (61.4)	257 (29.5)	87 273 (61.6)	
65‐74, n (%)	32 743 (22.9)	272 (31.3)	32 471 (22.9)	
≥75, n (%)	22 363 (15.7)	341 (39.2)	22 022 (15.5)	
Laterality				.012
Right, n (%)	69 932 (49.0)	421 (48.4)	69 511 (49.0)	
Left, n (%)	72 560 (50.9)	447 (51.4)	72 113 (50.9)	
Bilateral, n (%)	97 (0.07)	0 (0)	97 (0.07)	
Unknown, n (%)	47 (0.04)	2 (0.22)	45 (0.03)	
Combined stage, n (%)				<.001
Stage 0	17 722 (12.4)	70 (8.1)	17 652 (12.5)	
Stage I	56 087 (39.3)	305 (35.1)	55 782 (39.4)	
Stage II	46 317 (32.5)	278 (31.9)	46 039 (32.5)	
Stage III	15 117 (10.6)	164 (18.9)	14 953 (10.6)	
Stage IV	7393 (5.2)	53 (6.1)	7340 (5.2)	
Breast surgery, n (%)	130 719 (91.7)	772 (88.7)	129 947 (91.7)	.002
Radiation therapy, n (%)	62 245 (43.6)	139 (15.9)	62 106 (43.8)	<.001
Hormone therapy, n (%)	95 602 (67.0)	680 (78.2)	94 922 (66.9)	<.001
Chemotherapy, n (%)	53 210 (37.3)	236 (27.1)	52 974 (37.4)	<.001
Trastuzumab, n (%)	17 337 (12.2)	52 (5.9)	17 285 (12.2)	<.001

### 3.2 Differences in practice patterns for MBC and FBC patients with stages 0–III diseases

#### 3.2.1 Breast surgery

In patients with nonmetastatic diseases (stages 0–III) (N = 135,243), mean age of MBC patients was significantly higher than that of FBC patients (70 *vs* 60 years; *P* < .001). In both genders, >90% of the patients underwent breast surgery. On the other hand, among patients who received breast surgery, MBC patients underwent mastectomy more frequently than FBC patients (84.1% *vs* 46.6%; *P* < .001).

#### 3.2.2 Adjuvant radiation therapy

**Figure 1:**
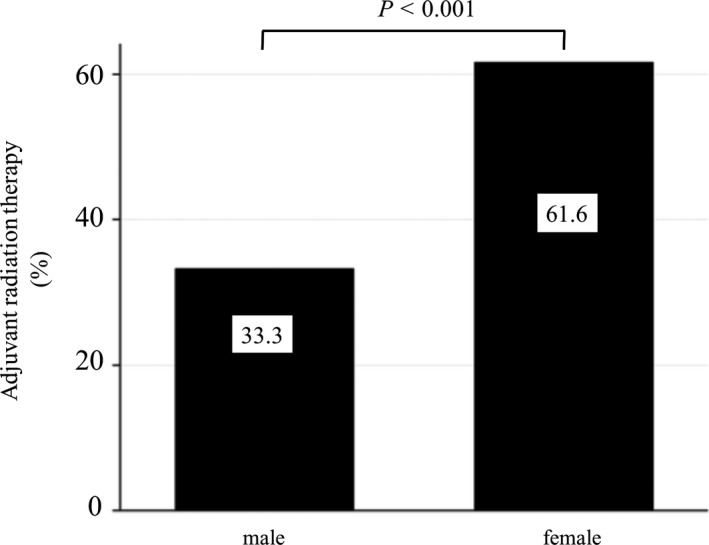
Differences in adjuvant radiation therapy between MBC and FBC patients The left bar indicates the MBC group, and the right bar indicates the FBC group. Among stage 0–III postsurgical patients who had a tumor size ≥ 5 cm or the number of positive axillary lymph nodes ≥ 4 after mastectomy, or who underwent lumpectomy for adjuvant radiation therapy (N = 52,894), MBC patients received adjuvant radiation therapy less frequently than FBC patients (33.3% *vs* 61.6%; *P* < .001). MBC; male breast cancer, FBC; female breast cancer

A total of 207 (0.39%) MBC patients received adjuvant radiation therapy among candidate patients (N = 52 894). The mean age and combined stage were higher in MBC patients than in FBC patients. Mastectomy was the preferred therapy among MBC patients (60.9% *vs* 21.8%; *P* < .001). MBC patients received adjuvant radiation therapy less frequently than FBC patients (33.3% *vs* 61.6%; *P* < .001; Figure 1). Even after adjusting for age, combined stage, and surgical method (mastectomy or lumpectomy), gender was an independent factor affecting the administration of adjuvant radiation therapy (OR 0.61, 95% CI 0.45‐0.84; *P* = .002; Table 2).

**Table 2:** Factors related to administration of adjuvant radiation therapy for stage 0–III postsurgical patients who had a tumor size ≥5 cm or the number of positive axillary lymph nodes ≥4 after mastectomy, or who underwent lumpectomy for adjuvant radiation therapy (N = 52 894)

Characteristics	Unadjusted Odds ratio (95% CI)	*P*‐value	Adjusted Odds ratio (95% CI)^a^	*P*‐value
Gender (male)	0.31 (0.23‐0.42)	<.001	0.61 (0.45‐0.84)	.002
Lumpectomy	3.64 (3.48‐3.79)	<.001	5.11 (4.80‐5.43)	<.001
Age, years				
≤64	Reference		Reference	
65‐74	0.92 (0.89‐0.96)	<.001	0.91 (0.87‐0.95)	<.001
≥75	0.29 (0.27‐0.31)	<.001	0.27 (0.25‐0.28)	<.001
Combined stage				
Stage 0	Reference		Reference	
Stage I	1.20 (1.13‐1.28)	<.001	1.34 (1.26‐1.42)	<.001
Stage II	0.75 (0.71‐0.80)	<.001	1.17 (1.10‐1.25)	<.001
Stage III	0.56 (0.52‐0.59)	<.001	2.24 (2.05‐2.45)	<.001

^a^ Adjusted for Age, Gender, Combined stage, Lumpectomy.

#### 3.2.3 Adjuvant hormone therapy of MBC and FBC patients

Of 129,144 who received adjuvant hormone therapy, 759 (0.59%) were MBC patients. Adjuvant hormone therapy was more frequently administered to MBC patients than to FBC patients (79.1% *vs* 67.6%; *P* < .001; Table S1). The former received tamoxifen twice as frequently as the latter (86.5% *vs* 41.2%; *P* < .001). Alternatively, aromatase inhibitors and gonadotropin‐releasing agonists were much less frequently administered to MBC patients than to FBC patients (16.7% *vs* 61.1%; *P* < .001, 2.2% *vs* 12.0%; *P* < .001, respectively).

#### 3.2.4 Neo‐adjuvant/adjuvant chemotherapy and adjuvant trastuzumab

**Figure 2:**
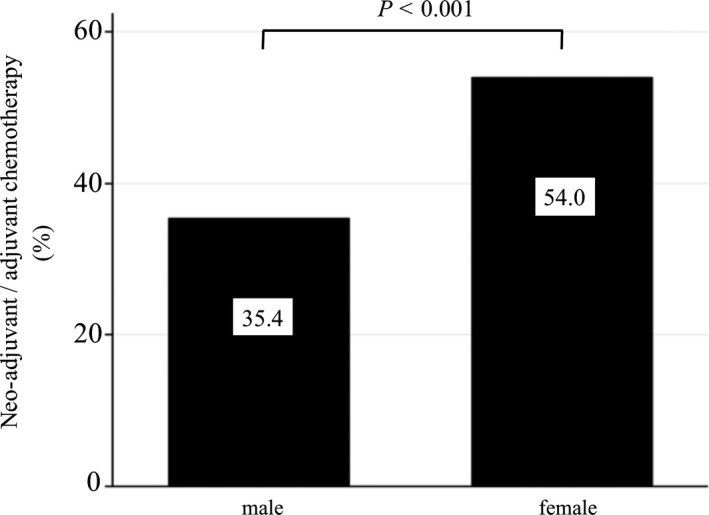
Differences in neo‐adjuvant/adjuvant chemotherapy between MBC and FBC patients indicated for these therapies (N = 57,550) The left bar indicates the MBC group, and the right bar indicates the FBC group. Among stage II‐III patients who received surgery, MBC patients received chemotherapy less frequently than FBC patients in neo‐adjuvant or adjuvant settings (35.4% *vs* 54.0%; *P* < .001). MBC; male breast cancer, FBC; female breast cancer

Stage II‐III patients who underwent surgery were considered potential candidates for adjuvant chemotherapy and trastuzumab therapy (N = 57,550). Among these, 395 (0.69%) were MBC patients and their mean age as well as the proportion of stage III patients was higher than that of FBC patients. Furthermore, chemotherapy was less frequently administered to MBC patients than FBC patients (35.4% *vs* 54.0%; *P* < .001; Figure 2). Of all MBC patients who received chemotherapy, 12.1% received an anthracycline‐based regimen, 50.7% received anthracycline‐based regimen followed by taxane, 27.9% a taxane‐based regimen, and 9.3% received another regimen. MBC patients received anthracycline‐based regimen followed by taxane (50.7% *vs* 64.5%; *P* = .001) and adjuvant trastuzumab (8.1% vs 18.1%; *P* < .001) much less frequently than FBC patients. The adjusted factors affecting chemotherapy in a logistic regression analysis for choosing to receive neo‐adjuvant/adjuvant chemotherapy are listed in Table 3. After adjusting for age and combined stage, gender remained an independent factor affecting decisions about administering neo‐adjuvant/adjuvant chemotherapy (OR 0.67, 95% CI 0.53‐0.85; *P* = .001; Table 3).

**Table 3:** Factors related to administration of neo‐adjuvant/adjuvant chemotherapy based on a logistic regression model among stage II‐III patients who underwent surgery (N = 57 550)

Characteristics	Unadjusted Odds ratio (95% CI)	*P*‐value	Adjusted Odds ratio (95% CI)^a^	*P*‐value
Gender (male)	0.47 (0.38‐0.57)	<.001	0.67 (0.53‐0.85)	.001
Age, years				
≤64	Reference		Reference	
65‐74	0.54 (0.52‐0.56)	<.001	0.53 (0.51‐0.55)	<.001
≥75	0.12 (0.11‐0.12)	<.001	0.11 (0.10‐0.11)	<.001
Combined stage				
Stage II	Reference		Reference	
Stage III	2.07 (1.99‐2.15)	<.001	2.36 (2.26‐2.47)	<.001

^a^ Adjusted for Age, Gender, Combined stage.

### 3.3 Differences in treatment for metastatic diseases

Among patients with metastatic diseases (stage IV, N = 7,393), the mean age of MBC patients was slightly higher than that of FBC patients (66 *vs* 61 years; *P* < .001). Compared with FBC patients, MBC patients received hormone therapy more frequently (73.6% *vs* 60.5%; *P* = .052), with a preference for tamoxifen (76.9% *vs* 34.4%; *P* < .001; Table S2), but received chemotherapy (52.8% *vs* 62.3%; *P* = .159) and trastuzumab (16.9% vs 23.1%; *P* = .29) less often. In logistic regression analysis, after adjusting for age, gender was not found to be a significant factor for choosing chemotherapy (OR 0.69, 95% CI 0.40‐1.20; *P* = .19).

## 4 DISCUSSION

Using a large, nationwide database, we demonstrated several important characteristics of MBC patients. First, MBC patients comprised 0.6% of all breast cancer patients, and were 10 years older than FBC patients. Second, although cancer stages were more advanced in MBC than in FBC patients, the former had a lower probability of receiving adjuvant radiation therapy and neo‐adjuvant/adjuvant chemotherapy. Third, hormone therapy was more frequently administered in MBC, than in FBC patients, whereas anti‐HER2 therapy was rarely selected for MBC patients.

Our results are consistent with studies published in other jurisdictions outside Japan. MBC patients are older at diagnosis, receive hormone therapy more frequently and anti‐HER2 therapy less frequently than FBC patients.^2^, ^19^, ^20^ A large, population‐based study showed that MBC patients had a strikingly higher rate (90.6%) of estrogen receptor‐positive tumors than FBC patients (76%),^2^ which supports gender differences in treatment patterns. Although no randomized controlled trials (RCT) assessed adjuvant tamoxifen for MBC patients, a single‐center, retrospective, observational study showed that adjuvant tamoxifen alleviated disease and ensured overall survival in MBC patients.^21^ However, the role of an aromatase inhibitor and gonadotropin‐releasing agonist in an adjuvant setting has not been established.^16^, ^22^ We showed that MBC patients received adjuvant hormone therapy and tamoxifen at a higher rate and aromatase inhibitor at a lower rate than FBC patients.

While our study showed that MBC patients received anti‐HER‐2 therapy less often than FBC patients, recent studies have shown that 15% of MBC patients and 20%–30% for FBC patients overexpress HER2.^4^, ^23^ Although data showing the efficacy of trastuzumab specifically for MBC are lacking, FBC patients show an established clinical benefit, and adjuvant trastuzumab is recommended for high‐risk HER2‐positive MBC patients. Since the database lacked pathological HER2 status, we were unable to assess whether HER2‐positive MBC patients received adjuvant trastuzumab therapy. Nevertheless, our results may reflect the HER2 status in MBC because anti‐HER2 therapy is likely indicated only for HER2‐positive patients.

We found that stage 0–III MBC patients (postsurgical MBC patients who are recommended adjuvant radiation therapy) were less likely to receive adjuvant radiation therapy compared to FBC patients. Although there is limited evidence about the indications for adjuvant radiation therapy in MBC patients, the indications generally recommended for MBC patients are similar to those recommended for FBC patients to prevent local recurrence.^15^, ^17^, ^24^ We acknowledge that several factors affect the decision on whether adjuvant radiation therapy is necessary in both MBC and FBC patients. However, even after adjusting for factors such as the type of surgical procedure, combined stage, and age, we showed that gender was a determinant of adjuvant radiation therapy. Future studies should address other reasons for a lower frequency of adjuvant radiation therapy for MBC patients.

Despite the robust evidence for adjuvant chemotherapy reducing the risk of recurrence in FBC patients, there are limited data, including the lack of RCTs, on the effectiveness of neo‐adjuvant/adjuvant chemotherapy in MBC. However, several retrospective study demonstrated the benefit of adjuvant chemotherapy for MBC patients,^25^, ^26^, ^27^ and one prospective study showed survival of 31 node‐positive stage II MBC patients who received cyclophosphamide, methotrexate, and 5‐fluorouracil (CMF) as adjuvant chemotherapy were 64.5% at 10‐years point, which was higher than that of Surveillance, Epidemiology, and End Results (SEER) reported data (42.5%).^28^ Thus, adjuvant chemotherapy should be considered for MBC patients, especially those who are node‐positive or have a higher‐stage disease.^4^

Regarding chemotherapy regimen, a previous study showed that an anthracycline‐based regimen improved disease‐free and overall survival.^29^ In general, anthracycline followed by taxane regimen has been used in MBC patients in the same manner as in FBC patients. Furthermore, the Japanese Breast Cancer Society Guidelines and NCCN guidelines recommend adjuvant chemotherapy for MBC patients with the same indications and the same regimen as for FBC patients.^15^, ^17^ Contrary to these recommendations, our study showed that MBC patients were less likely to receive neo‐adjuvant/adjuvant chemotherapy and less intensive regimens than FBC patients. When combined with our results on adjuvant radiation therapy, MBC patients undergo less optimal adjuvant therapy than FBC patients, which may be attributed to the lack of substantial evidence that support the efficacy of adjuvant radiation therapy and chemotherapy. Another reason may be the comparatively poor condition of male patients than female patients. It is possible that oncologists avoided prescribing adjuvant radiation therapy as well as adjuvant chemotherapy because of the general health condition of MBC patients.

The current study has some limitations. First, it was a retrospective study of voluntarily participating hospitals. Although the number of hospitals in the study was large, they may be highly motivated to improve the quality of care and may not be representative of all Japanese hospitals. Second, information on biomarkers, such as hormone receptor status and HER2 overexpression, was not available in our study. This limited our ability to determine the reasons for differences in practice patterns regarding hormone and trastuzumab therapies between MBC and FBC patients, which may be attributed to differences in the distribution of biomarker profiles between genders. Ideally, a prospective study covering these important factors is desirable to assess appropriate treatments. However, the rarity of MBC is a challenge for patient recruitment. Therefore, retrospective longitudinal studies similar to this study are best suited to describe practice patterns and patient characteristics.

In conclusion, we successfully used a large database to describe the epidemiology and practice patterns of MBC. We identified that MBC patients are extremely rare, comprising 0.6% of all breast cancer patients, and were on average 10 years older than FBC patients. Despite being diagnosed with more advanced disease than FBC patients, adjuvant therapies, including radiation and chemotherapy, were less likely to be administered to MBC patients compared to FBC patients. Due to the rarity of MBC, studies on MBC patients may be complicated; nevertheless, more detailed studies are required to ensure appropriate delivery of care.

## CONFLICT OF INTEREST

The authors have declared no conflicts of interest.

## AUTHOR CONTRIBUTIONS

All authors contributed to the study conception and design. Material preparation, data collection, and analysis were performed by Taisuke Ishii, Tomone Watanabe and Takahiro Higashi. The first draft of the manuscript was written by Taisuke Ishii and all authors commented on previous versions of the manuscript. All authors read and approved the final manuscript.

## Supporting information

Table S1‐S2

## Data Availability

Data sharing is not applicable to this article as no new data were created or analyzed in this study.
