# Supplementary material for: Epidemiology and practice patterns for male breast cancer compared with female breast cancer in Japan
Source: Cancer Med. 2020 Jul 1;9(16):6069–75. doi: 10.1002/cam4.3267 (PMC7433825; doi:10.1002/cam4.3267)
Supplement: Supplementary file 1 — Table S1‐S2 [file CAM4-9-6069-s001.docx]

Table S1. Differences in adjuvant hormone therapy between male and female among post-surgical stage 0–III breast cancer patients (*N* = 129,144)

| Characteristics | Male  (n = 759) | Female  (n = 128,385) | *p*-value* |
| --- | --- | --- | --- |
| Hormone therapy, n (%) | 600 (79.1) | 86,775 (67.6) | <0.001 |
| Tamoxifen, n (%) | 519 (86.5) | 35,766 (41.2) | <0.001 |
| Aromatase inhibitor, n (%) | 100 (16.7) | 53,053 (61.1) | <0.001 |
| GnRH agonist, n (%) | 13 (2.2) | 10,419 (12.0) | <0.001 |

* Statistical test for the difference between male and female patients

GnRH agonist; gonadotropin-releasing hormone agonist

Table S2. Differences in treatment between male and female patients with stage IV breast cancers (*N* = 7,393)

| Characteristics | Male  (n = 53) | Female  (n = 7,340) | *p*-value* |
| --- | --- | --- | --- |
| Breast surgery, n (%) | 17 (31.3) | 2,297 (31.3) | 0.90 |
| Hormone therapy, n (%) | 39 (73.6) | 4,441 (60.5) | 0.052 |
| Tamoxifen, n (%) | 30 (76.9) | 1,526 (34.4) | <0.001 |
| Aromatase inhibitor, n (%) | 17 (43.6) | 3,478 (78.3) | <0.001 |
| GnRH agonist, n (%) | 3 (7.7) | 698 (15.7) | 0.17 |
| MPA, n (%) | 1 (2.6) | 148 (3.3) | 0.79 |
| Fulvestrant, n (%) | 6 (15.4) | 662 (14.9) | 0.93 |
| Chemotherapy, n (%) | 28 (52.8) | 4,569 (62.3) | 0.159 |
| Trastuzumab, n (%) | 9 (16.9) | 1,693 (23.1) | 0.29 |

* Statistical test for the difference between male and female patients

GnRH agonist; gonadotropin-releasing hormone agonist, MPA; medroxyprogesterone acetate
